# Anti-Cancer Effects of CKD-581, a Potent Histone Deacetylase Inhibitor against Diffuse Large B-Cell Lymphoma

**DOI:** 10.3390/ijms21124377

**Published:** 2020-06-19

**Authors:** Soo Jin Kim, U Ji Kim, Hae Yong Yoo, Yong June Choi, Keon Wook Kang

**Affiliations:** 1 College of Pharmacy and Research Institute of Pharmaceutical Sciences, Seoul National University, Seoul 08826, Korea; kimsj@ckdpharm.com (S.J.K.); cyjune112@snu.ac.kr (Y.J.C.); 2 CKD Research Institution, Chong Kun Dang Pharmaceutical Corporation, Gyeonggi-do 16995, Korea; ujik@ckdpharm.com; 3 Department of Health Sciences and Technology, Samsung Advanced Institute for Health Sciences and Technology, Samsung Medical Center, Sungkyunkwan University, Seoul 08826, Korea; haeyong.yoo@samsung.com

**Keywords:** CKD-581, diffuse large B cell lymphoma, histone deacetylase inhibitor, MYC

## Abstract

Double-hit lymphoma (DHL) and double-expressor lymphoma (DEL) are aggressive forms of lymphoma that require better treatments to improve patient outcomes. CKD-581 is a new histone deacetylase (HDAC) inhibitor that exhibited a better safety profile in clinical trials compared to other HDAC inhibitors. Here, we demonstrate that CKD-581 inhibited the class I–II HDAC family via histone H3 and tubulin acetylation. CKD-581 treatment also up-regulated the phosphorylation of histone H2AX (γH2AX, DNA double-strand break marker), and reduced levels of MYC and anti-apoptotic proteins such as BCL-2, BCL-6, BCL-_X_L, and MCL-1 in DH/DE-diffuse large B cell lymphoma (DLBCL) cell lines. Ultimately, CKD-581 also induced apoptosis via poly(ADP ribose) polymerase 1 (PARP1) cleavage. In a DLBCL SCID mouse xenograft model, CKD-581 exhibited anti-cancer effects comparable with those of rituximab (CD20 mAb). Our findings suggest that CKD-581 could be a good candidate for the treatment of DLBCL.

## 1. Introduction

Diffuse large B cell lymphoma (DLBCL) is the most common subtype of lymphoma, accounting for approximately 30% of all lymphoma [1]. DLBCL can be further classified into activated B-cell (ABC)- and germinal center-B cell (GCB)-DLBCL depending on the cellular origin. Of these two subtypes, patients with ABC-DLBCL have worse outcomes [2]. 

The standard chemotherapeutic regimen for DLBCL is rituximab, cyclophosphamide, doxorubicin, vincristine, and prednisone (R-CHOP). About 60–70% of patients are cured by R-CHOP therapy; the remaining 30–40% of patients respond poorly to standard therapy [3]. Among the patients who failed treatment with R-CHOP therapy, 20% suffered from primary refractory disease and 30% relapsed after achieving a complete response (CR) [4]. Most refractory patients exhibited double-hit lymphoma (DHL) or double-expresser lymphoma (DEL) [4]. Ultimately, patients who fail standard therapy have limited treatment options [5].

MYC is a representative proto-oncogene that is overexpressed in lymphomas [6]. MYC rearrangement is frequently associated with BCL-2 and/or BCL-6 translocation [7], and influences whether DLBCL is classified as DHL or triple-hit lymphoma (THL) [7,8]. Concomitant overexpression of MYC and BCL-2 without chromosomal rearrangement occurs in 20%~35% of DLBCL patients [7]. This subtype is referred to as DEL (double-expresser lymphoma) [6]. Since DHL, THL, and DEL are aggressive B-cell lymphomas with poor prognoses [9,10], there is an urgent unmet clinical need to identify more effective therapies [11].

Histone deacetylase (HDAC) inhibitors are epigenetic drugs that modify gene expression, restoring the normal differentiation and death program usually altered in cancer cells [12]. HDAC inhibitors have been approved for the treatment of lymphoma and multiple myeloma (MM). For example, vorinostat was approved for treating cutaneous T-cell lymphoma (CTCL), while romidepsin was approved for treating CTCL and peripheral T-cell lymphoma (PTCL), belinostat for treating PTCL, and panobinostat for treating MM [13]. Although several HDAC inhibitors are under investigation for the treatment of DLBCL, these compounds, including vorinostat, belinostat, and panobinostat, showed limited activities or unexpected toxicities in clinical trials [14]. Romidepsin showed anti-cancer activity via BCL-6 suppression in lymphoma, and now the compound is in clinical trials for combination therapies with various agents [15].

However, no HDAC inhibitor has been approved for treating DLBCL, despite the urgent need for an appropriate chemotherapeutic regimen. CKD-581 (alteminostat) is a new HDAC inhibitor that targets class I–II HDACs. A phase I clinical study was conducted to evaluate the safety and tolerability of CKD-581 for patients with Hodgkin lymphoma, non-Hodgkin lymphoma (mantle cell lymphoma, DLBCL, PTCL, marginal zone lymphoma, Burkitt’s lymphoma, follicular lymphoma), and refractory MM. CKD-581 was well tolerated, and 16 of 36 (44.4%) patients achieved a stable disease (SD) or better [16].

In this study, the in vitro and in vivo anti-cancer activities of CKD-581 were assessed in four different DLBCL cell types. The pharmacological mechanism was investigated with a focus on the regulation of MYC and the anti-apoptotic BCL-2 family.

## 2. Materials and Methods

### 2.1. Antibodies and Reagents

CKD-581 was provided from CKD Pharmaceutical Corp. (Seoul, Korea) and suberoylanilide hydroxamic acid (SAHA, vorinostat) with a purity of >98% was purchased from Sigma-Aldrich (St. Louis, MO, USA). Rituximab was purchased from Roche (Basel, Switzerland). Compounds were dissolved in dimethyl sulfoxide (DMSO) and stored at −20 °C. The CellTiter-Glo luminescent cell viability assay kit was supplied from Promega (Madison, WI, USA). Antibodies recognizing histone H3, acetylated histone H3, tubulin, acetylated tubulin, myeloid cell leukemia-1 (MCL-1), and poly (ADP-ribose) polymerase 1 (PARP1) were purchased from Cell Signaling Technology (Danvers, MA, USA). Antibodies for BCL-2, BCL-6, and BCL-_X_L were obtained from Santa Cruz Biotechnology (Dallas, TX, USA). Antibody for phosphorylated H2AX (γH2AX) was purchased from Abcam (Cambridge, MA, USA). Antibodies for MYC, CD20, and glyceraldehyde 3-phosphate dehydrogenase (GAPDH) were supplied from Thermo Fisher Scientific (Waltham, MA, USA). 

### 2.2. Cell Viability Assay

Human DLBCL cell lines, SU-DHL-2 (ABC/DH), and SU-DHL-4 (GCB/DH) were purchased from American Type Culture Collection (ATCC, Rockville, MD, USA). OCI-LY1 (GCB/DH) and U2932 (ABC/DE) cell lines were kindly donated by Dr. Yu HY (Samsung Medical Center, Seoul, Korea). SU-DHL-2, SU-DHL-4, and U2932 cells were cultured in RPMI1640 (Thermo Fisher Scientific) containing 10% fetal bovine serum (Thermo Fisher Scientific) at 37 °C in 5% CO_2_. OCI-LY1 cells were cultured in IMDM (Thermo Fisher Scientific), containing 20% fetal bovine serum. All cells were seeded in a 96-well plate and allowed to incubate overnight, then treated with serial dilutions of CKD-581 or SAHA. After a 72-h incubation, cell viability was assessed by the CellTiter Bright-Glo system (Promega). Relative viable cell percentages were quantified by measuring luminescence using a Glomax luminometer (Promega). 

### 2.3. Histone Protein Extraction

SU-DHL-2 cells were incubated with CKD-581 or DMSO for 6 h, harvested, and washed twice with phosphate-buffered saline (PBS). The cells were suspended with 50 μL ice-cold lysis buffer (10 mM Tris-HCl (pH 6.5), 50 mM sodium bisulfate, 1% Triton X-100, 10 mM MgCl_2_, and 8.6% sucrose) and centrifuged at 1500× *g* for 10 min at 4 °C. Pelleted nuclei were suspended with 50 μL 0.4 *N* H_2_SO_4_, and incubated on ice for 1 h, then centrifuged at 1500× *g* for 10 min at 4 °C. The supernatant proteins were precipitated with 500 μL ice-cold acetone overnight at −20 °C. The precipitated proteins were collected by centrifugation at 1000× *g* for 10 min at 4 °C, air dried, and suspended in 50 μL deionized water.

### 2.4. Western Blot Analysis

Cells were lysed with a RIPA lysis buffer (Sigma-Aldrich) supplemented with protease and phosphatase inhibitor cocktails (Roche). Total cell lysates were resolved on 4–12% gradient SDS-PAGE gel (Thermo Fisher Scientific) and transferred to a nitrocellulose membrane. After blocking with 5% skim milk in Tris-buffered saline containing 0.05% Tween 20 (TBS-T), the membrane was incubated with primary and HRP-conjugated secondary antibodies. Chemiluminescence signals were detected by Chemidoc (BioRad, Hercules, CA).

### 2.5. Xenograft Study

Male NOD.CB17 SCID mice were subcutaneously implanted with various human B cell lymphoma cell lines. After tumor volumes reached 100–200 mm^3^, the animals were randomized into treatment groups, including vehicle (0.9% saline, twice a week), CKD-581 (20 or 40 mg/kg, twice a week), and rituximab (10 mg/kg, once a week). All the mice were intraperitoneally injected with vehicle or treatment agents. Tumors and body weights were measured 2 times a week. Tumor volume was monitored using external measurements with a caliper and tumor volumes were calculated using the formula (width^2^ × length)/2.

### 2.6. Statistical Analysis 

One-way ANOVA was used to determine the differences in all experiments except the tumor xenograft experiment. For the tumor xenograft experiment, two-way ANOVA was used; significance was expressed as * *p* < 0.05, ** *p* < 0.01, and *** *p* < 0.001. All data were analyzed using GraphPad Prism software (Irvine, CA, USA).

## 3. Results

### 3.1. CKD-581 Enhances Acetylation of HDAC Target Proteins

**Figure 1:**
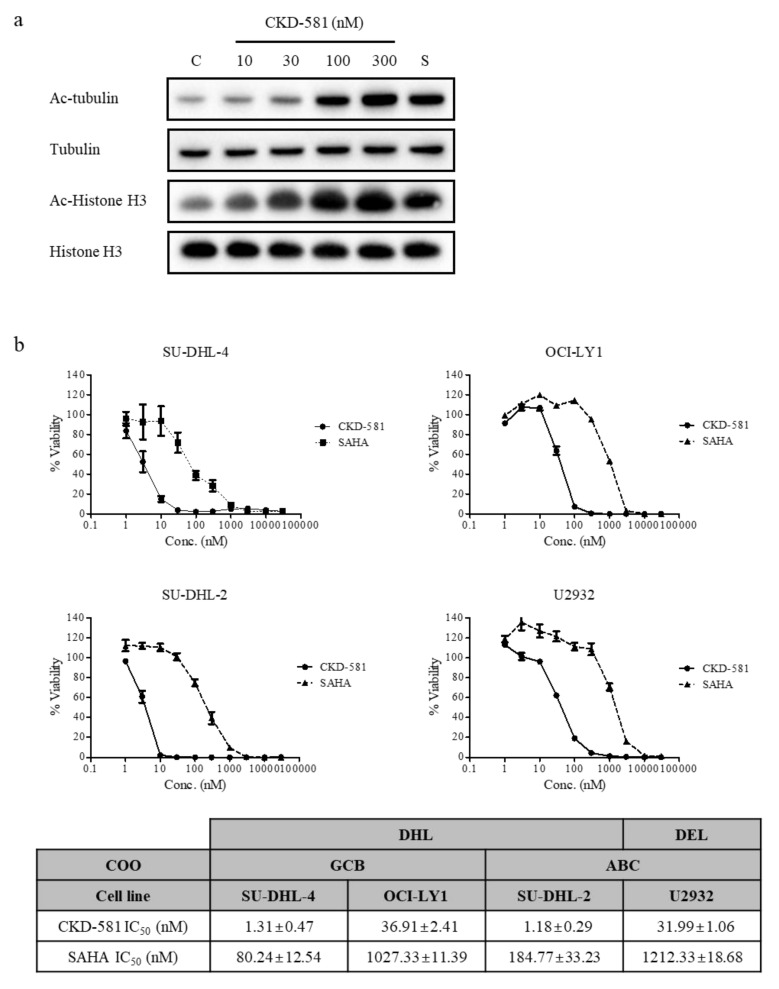
CKD-581 is a potent HDAC inhibitor. (**a**) Acetylation of tubulin or histone H3. SU-DHL-2 cells were treated with vehicle control (C), CKD-581 (10–300 nM), or 300 nM SAHA (S) for 6 h, and total cell lysates were obtained. Acetylation of tubulin or histone H3 was determined by immunoblotting. (**b**) Comparison of inhibitory effects of CKD-581 and SAHA on cell viability of four DLBCL cell lines. SU-DHL-4, OCI-LY1, SU-DHL-2, and U2932 cells were incubated with CKD-581 and SAHA for 72 h, and cell viability was assessed by a CellTiter Bright-Glo assay. Data represent mean ± SEM (*n* = 3).

Histone H3 and tubulin are representative target proteins of HDACs. The acetylation status of either protein can be affected by HDAC inhibitors. It is well established that acetylated histone proteins are regulated by HDAC class I in the nucleus, and that acetylated tubulin is a target of HDAC class II in cytoplasm [17]. The acetylation of histone H3 in SU-DHL-2 cells increased following CKD-581 (30–300 nM) treatment, which was comparable to 300 nM SAHA. Moreover, tubulin acetylation also increased with 10 nM CKD-581 (Figure 1a). These results demonstrate that CKD-581 increased the acetylation of target molecules by inhibiting class I–II HDACs in lymphoma cells. 

### 3.2. CKD-581 Reduces Cell Viability of B-Cell Lymphoma Cell Lines 

The anti-proliferative effects of CKD-581 on four B-cell lymphoma cell lines were assessed using CellTiter Bright-Glo assays. Three DH-DLBCL cell lines (SU-DHL-2, SU-DHL-4, and OCI-LY1) and one DE-DLBCL cell line (U2932) [8,18] were tested. DH- and DE-DLBCL are aggressive non-Hodgkin lymphomas (NHLs) [9,10]. CKD-581 potently reduced cell viability in all four lymphoma cell lines in a concentration-dependent manner. The half maximal inhibitory concentration (IC_50_) values of CKD-581 in SU-DHL-4, OCI-LY1, SU-DHL-2, and U2932 cells were 1.31 ± 0.47, 36.91 ± 2.41, 1.18 ± 0.29, and 31.99 ± 1.06 nM, respectively. Based on the IC_50_ values of SAHA, CKD-581 was more potent in all DLBCL cell lines (Figure 1b). 

### 3.3. CKD-581 Decreases the Expression of Poor Prognostic Markers in Lymphoma Cells

**Figure 2:**
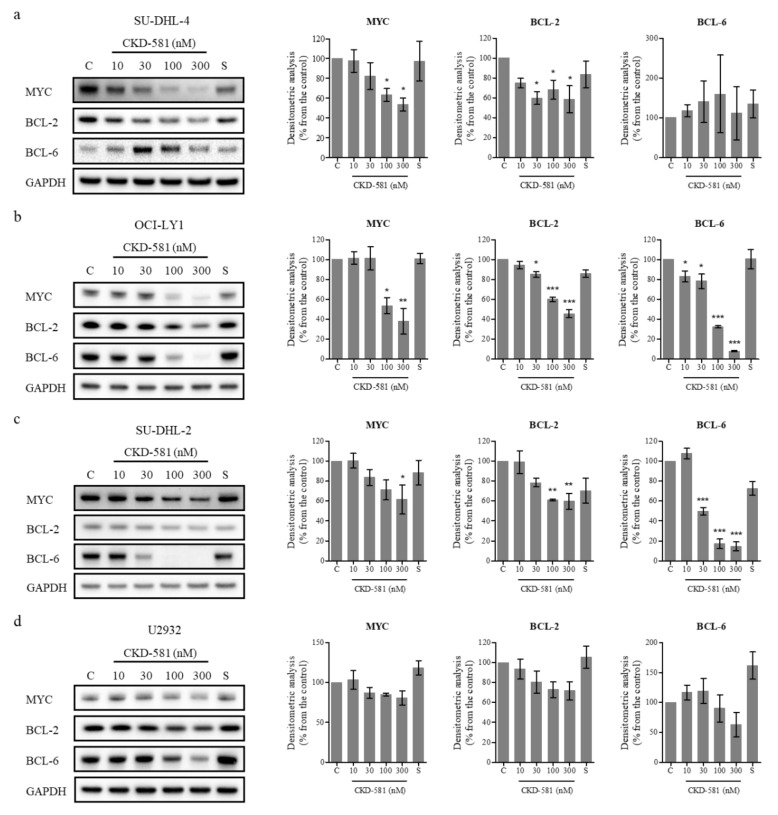
CKD-581 reduces the protein expression of prognostic markers for DLBCL. (**a**) SU-DHL-4, (**b**) OCI-LY1, (**c**) SU-DHL-2, and (**d**) U2932 cells were treated with vehicle control (C), various concentrations (10–300 nM) of CKD-581, or 300 nM SAHA (S) for 24 h, and total cell lysates were subjected to immunoblotting for MYC, BCL-2 and BCL-6. Data represent mean ± SEM of at least three independent experiments (*n* = 3, significant vs. control; * *p* < 0.05, ** *p* < 0.01, *** *p* < 0.001).

To investigate the effects of CKD-581 on the expression of poor prognostic factors, several molecules were identified by immunoblotting (Figure 2a–d). CKD-581 effectively decreased the expression of MYC and BCL-2 in the two GCB-DLBCL cell lines (Figure 2a,b). Although CKD-581 decreased the expression of BCL-6 as well as BCL-2 in OCI-LY1 cells, we did not find a significant reducing effect of CKD-581 on BCL-6 expression in SU-DHL-4 cells (Figure 2a,b). Moreover, the protein levels of MYC and BCL-6 were decreased by CKD-581 in ABC-DLBCL (Figure 2c,d). In comparison, the reference HDAC inhibitor, SAHA, only marginally reduced the expression of the poor prognostic factors of MYC, BCL-2, and BCL-6 in the tested lymphoma cell lines. The data indicate that CKD-581 efficiently decreased the expression of oncoproteins that are frequently expressed in DLBCL. In addition, considering the IC_50_ values of CKD-581 in each cell type, the inhibitory effects of CKD-581 on the expression of oncoproteins seem to be related to its anti-cancer effects. 

### 3.4. CKD-581 Induces DNA Damage in Lymphoma Cells

**Figure 3:**
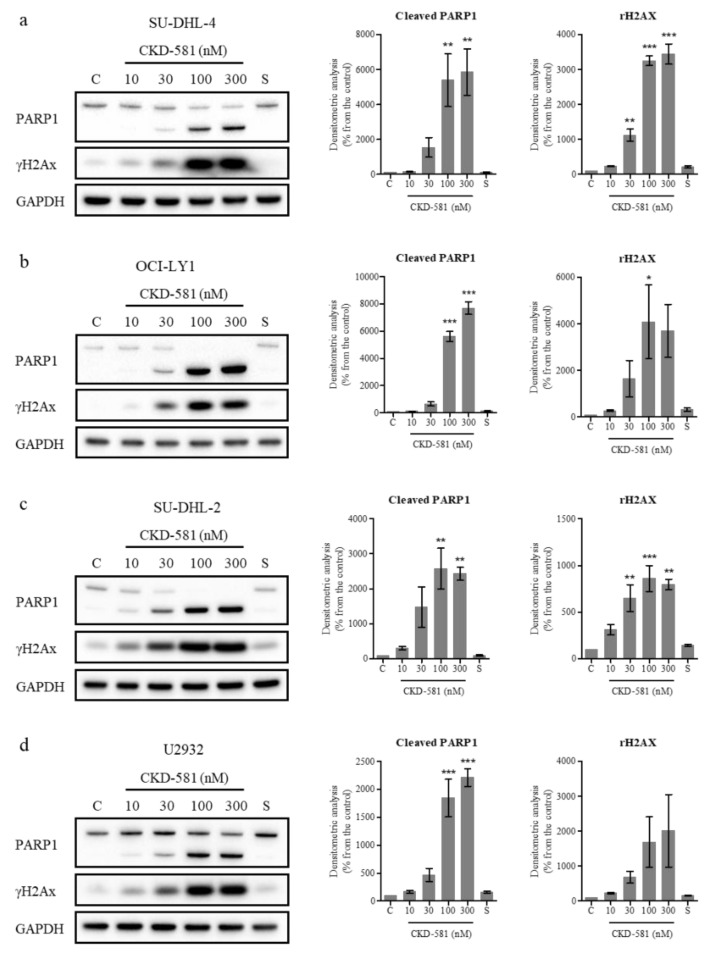
CKD-581 induces DNA damage and apoptosis. (**a**) SU-DHL-4, (**b**) OCI-LY1, (**c**) SU-DHL-2, and (**d**) U2932 cells were treated with vehicle control (C), CKD-581 (10–300 nM), or 300 nM SAHA (S) for 24 h, and total cell lysates were subjected to immunoblotting for γH2AX and PARP1. Data represent mean ± SEM (*n* = 3, significant vs. control; * *p* < 0.05, ** *p* < 0.01, *** *p* < 0.001).

The status of poly(ADP ribose) polymerase 1 (PARP1) and γH2AX (phosphorylated histone H2AX) was also assessed by immunoblotting. PARP1 cleavage can be triggered by DNA strand breaks (DSBs) and caspase-3-dependent cleavage. PARP1 is cleaved into 89 and 24 kDa fragments, which are representative markers of apoptosis [19]. γH2AX has also been regarded as a marker of DNA double-strand breaks [19]. In all types of DLBCL, CKD-581 (30‒300 nM) exposure for 24 h resulted in γH2AX accumulation and PARP1 cleavage (Figure 3a‒d). 

BCL-_X_L and MCL-1 are anti-apoptotic members of the BCL-2 family which are amplified in diverse cancer types [20]. Previous studies demonstrated that a loss of BCL-_X_L or MCL-1 delayed MYC-driven B cell lymphomagenesis [21,22]. On the contrary, overexpression of BCL-_X_L and MYC caused B-cell lymphoma, and overexpressed MCL-1 accelerated MYC-driven B-cell lymphoma development [23,24]. In addition, overexpression of BCL-_X_L and MCL-1 are known to be related to resistance to rituximab and other chemotherapies [25,26]. Hence, the down-regulation of BCL-_X_L and MCL-1 could be beneficial for treating B-cell lymphoma, including DLBCL. 

**Figure 4:**
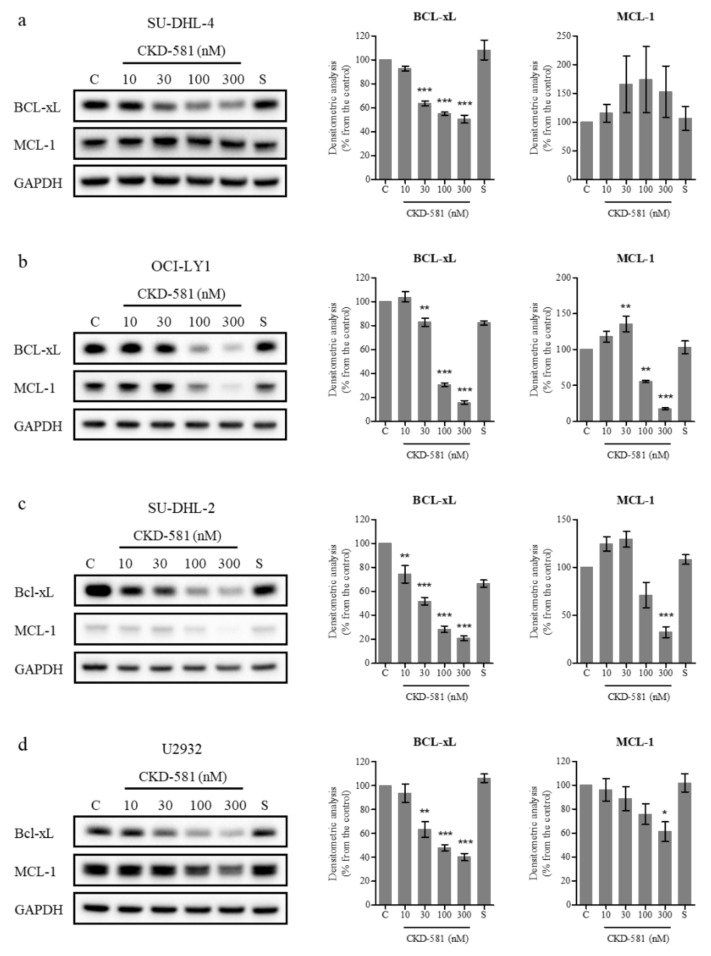
CKD-581 decreases anti-apoptotic proteins in DLBCL. (**a**) SU-DHL-4, (**b**) OCI-LY1, (**c**) SU-DHL-2, and (**d**) U2932 cells were treated with vehicle control (C), CKD-581 (10‒300 nM), or 300 nM SAHA (S) for 24 h, and total cell lysates were subjected to immunoblotting for BCL-xL and MCL-1. Data represent mean ± SEM (*n* = 3, significant vs. control; * *p* < 0.05, ** *p* < 0.01, *** *p* < 0.001).

Inhibitors targeting BCL-_X_L (AT-101, ABT-263) and MCL-1 (AZD5991, MIK665, AMG 176) have been developed for DLBCL and are undergoing clinical trials [27,28]. To determine the effect of these inhibitors on the cellular activity of BCL-2 family members, DLBCL cells were treated with various concentrations of CKD-581 for 24 h. For comparison, 300 nM SAHA was also investigated. BCL-_X_L expression was high in all four tested cell lines, as was the expression of MCL-1 in all cell lines, with the exception of SU-DHL-2 cells (Figure 4a–d). CKD-581 decreased the protein levels of BCL-_X_L and MCL-1 in a concentration-dependent manner (Figure 4b–d). Despite low levels of basal expression, the level of MCL-1 was also decreased by CKD-581 in SU-DHL-2 cells (Figure 4c). However, CKD-581 did not have any effect on MCL-1 expression in SU-DHL-4 cells (Figure 4a). This indicated that the anti-cancer effect of CKD-581 would be dependent on other BCL-2 family proteins, such as BCL-2 and BCL-_X_L, in SU-DHL-4 cells. These results suggest that CKD-581 stimulates the generation of DSBs in DLBCL, and shifts the balance from cell survival to apoptosis, which leads to cancer cell death.

### 3.5. CKD-581 Is Active in Animal Models of B-Cell Lymphoma 

**Figure 5:**
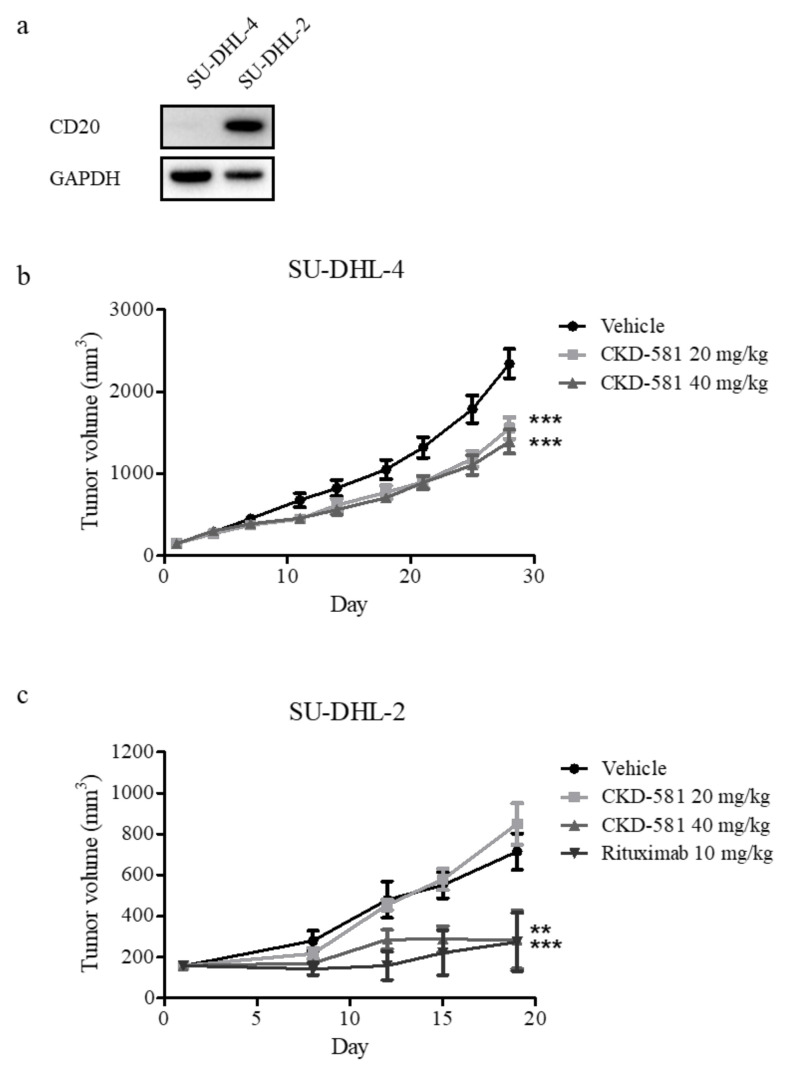
CKD-581 suppressed DH-DLBCL tumor growth in the mouse xenograft models. (**a**) CD20 expression in DH-DLBCL cells. CD20 expression levels were compared in SU-DHL-4 and SU-DHL-2 cells by immunoblotting. (**b**,**c**) Xenograft tumor growth assays. NOD.CB17 SCID mice were implanted with (**b**) SU-DHL-4 (1 × 10^6^ cells/mouse) or (**c**) SU-DHL-2 (1 × 10^6^ cells/mouse) cells. When tumors grew to about 150 mm^3^, the mice were intraperitoneally injected with vehicle, CKD-581 (20 and 40 mg/kg), or rituximab (10 mg/kg) according to the treatment schedule. Data represent mean ± SEM ((*n* = 10, SU-DHL-4; *n* = 6, SU-DHL-2) significant vs. vehicle group; * *p* < 0.05, ** *p* < 0.01, *** *p* < 0.001).

To assess the anti-cancer effects of CKD-581 in vivo, SU-DHL-4 and SU-DHL-2 xenograft mice were generated, given that these were the two cell types most sensitive to CKD-581. NOD.CB17 SCID mice implanted with both cell types received an intraperitoneal injection of vehicle or CKD-581 (20 and 40 mg/kg) twice a week. As shown in Figure 5a, CD20 was selectively expressed in SU-DHL-2 cells, but not in SU-DHL-4 cells. Thus, we further tested the anti-cancer effect of rituximab (CD20 monoclonal antibody (mAb), 10 mg/kg, once a week) as a positive control in the SU-DHL-2 xenograft model. In SU-DHL-4-implanted xenografts, CKD-581 partially but significantly suppressed tumor growth (Figure 5b). However, 40 mg/kg CKD-581 potently suppressed the growth of SU-DHL-2-implanted xenografts, to a degree comparable to 10 mg/kg rituximab (Figure 5c). 

## 4. Discussion

Rituximab (CD20 mAb) is an important therapeutic agent for NHL. Rituximab was effective as a single agent [29] and the addition of rituximab to CHOP (R-CHOP) improved the prognosis in DLBCL patients [30]. As a result, it was approved as first-line treatment for NHLs, including DLBCL [31]. However, 30–40% of DLBCL patients suffered from refractory or relapsed disease after an initial response to therapy [4]. Additionally, the anti-cancer activity of rituximab was limited in BCL-_X_L-expressing DLBCL [25]. Therefore, new treatments are needed for refractory/relapsed DLBCL patients. In this study, we investigated the anti-cancer effect of CKD-581 in DLBCL, and its potential mechanisms. 

CKD-581 had potent anti-proliferation effects against aggressive DLBCL, including DH- and DE-DLBCL. CKD-581 also down-regulated MYC and anti-apoptotic BCL-2 family members. Furthermore, it induced apoptosis, as shown by the presence of PARP1 cleavage in aggressive NHLs. Finally, CKD-581 had comparable in vivo anti-cancer efficacy to rituximab in CD20 expressing DLBCL (Figure 5b). Hence, CKD-581 was effective regardless of CD20 and/or BCL-_X_L expression, and would be a candidate for clinical treatment of lymphoma, especially refractory/relapsed lymphoma after rituximab-based therapy. As shown in Figure 2d, MYC reduction was marginal in CKD-581-treated U2932 cells (DEL). However, the IC_50_ value of cancer cell death was similar to those of other cell lines (Figure 1), implying that the anti-cancer activity of CKD-581 may have resulted not only from MYC reduction, but other mechanisms such as DNA damage and decreased expression of anti-apoptotic proteins in DLBCL cell lines.

As a new HDAC inhibitor, the safety, pharmacokinetics, and pharmacodynamics of CKD-581 were assessed in a Phase 1 clinical study [16]. Although thrombocytopenia is a common side effect of HDAC inhibitors [32,33], CKD-581 showed a relatively lower incidence of hematological toxicity compared to other HDAC inhibitors. Among a total of 39 patients, Grade 3 neutropenia (1 patient at 50 mg/m^2^) and Grade 4 thrombocytopenia (2 patients at 210 mg/m^2^) were reported [16].

Cardiac toxicity is another important safety concern for HDAC inhibitors [34,35]. However, cardiac toxicities such as QTc prolongation and arrhythmias were not observed in a clinical study of CKD-581 [16]. Moreover, CKD-581 yielded dose-proportional increases in area under the curve (AUC) and C_max_ values, which were distinct from the non-linear PK profiles of other HDAC inhibitors [36,37]. Considering that CKD-581 has a better pharmacokinetic and toxicity profile compared to other HDAC inhibitors, it is a leading therapeutic candidate for DLBCL treatment.

To improve the clinical outcomes of cancer, combination therapy is widely applied because single agents have insufficient efficacy, where resistance to therapy is likely to emerge [38,39]. Although CKD-581 showed potent anti-cancer effects in cell-based and xenograft assays using DLBCL cell lines, its pharmacological efficacy is not sufficient for clinical use. The clinical efficacy of CKD-581 could be improved by use in combination with other agents that have different mechanisms. 

For instance, bortezomib could be suitable for combination with CKD-581. It has also been reported that HDAC inhibitors enhance the accumulation of misfolded proteins induced by proteasome inhibitors [40,41]. Bruton’s tyrosine kinase (BTK) inhibitor has also been proposed as a combination therapy to HDAC inhibitor treatment, to overcome acquired resistance in lymphoma [42]. HDAC inhibitor upregulated the B-cell receptor (BCR) pathway through the upregulation of IRE1, which occurred due to the unfolded protein response (UPR). A combination of an HDAC inhibitor and a BTK inhibitor inhibited UPR and BCR pathways, which reduced tumor growth. Therefore, CKD-581 has potential for wide use in combination with various types of anti-cancer agents for the treatment of DLBCL.

## 5. Conclusions

CKD-581 is a new broad-spectrum HDAC inhibitor that targets type I and II HDACs. This investigation demonstrated for the first time that CKD-581 reduced the expression of MYC and anti-apoptotic BCL-2 family members in a concentration-dependent manner. Moreover, the expression levels of important prognostic markers for DLBCL, including BCL-2, BCL-6, BCL-_X_L, and MCL-1, were all reduced. CKD-581 also induced apoptosis in aggressive DLBCL cell lines and showed outstanding in vivo anti-cancer activity in aggressive DLBCL. Despite many efforts to develop new regimens for DLBCL, there are currently no HDAC inhibitors approved for this indication. Because CKD-581 is an efficacious HDAC inhibitor that maintains a proper safety profile, CKD-581 could be a promising drug candidate for the treatment of DLBCL.
